# Determinants of influenza vaccination uptake in pregnancy: a large single-Centre cohort study

**DOI:** 10.1186/s12884-019-2628-5

**Published:** 2019-12-19

**Authors:** Stéphanie Bartolo, Emilie Deliege, Ophélie Mancel, Philippe Dufour, Sophie Vanderstichele, Marielle Roumilhac, Yamina Hammou, Sophie Carpentier, Rodrigue Dessein, Damien Subtil, Karine Faure

**Affiliations:** 1 University Lille, EA 2694 : épidémiologie et qualité des soins, pôle recherche aile Est 2ème étage, 59 045 cedex, 1 Place de Verdun, 59 000 Lille, France; 2 Douai hospital, route de Cambrai, -, 10740 - 59507 Douai Cedex, BP France; 3 University Lille, CHU Lille, Pôle Femme Mère Nouveau-né, Avenue Eugène Avinée, 59000 Lille, France; 4 University Lille, EA7366, Recherche Translationelle Relation Hôte-Pathogènes, Faculté de Médecine Pôle Recherche 5 ème étage Ouest, 1 Place de Verdun, 59045 Lille, France; 5 University Lille, CHU Lille, Service de Maladies Infectieuses, rue Michel Polonowski, 59000 Lille, France

**Keywords:** Influenza vaccine, Pregnancy, Health knowledge, Behaviours

## Abstract

**Background:**

Although vaccination of pregnant women against influenza is recommended, the vaccination rate remains low. We conducted a study to identify determinants of influenza vaccination uptake in pregnancy in order to identify strategies to improve seasonal influenza vaccination rates.

**Methods:**

Prospective observational hospital-based study in the French hospital performing the highest number of deliveries, located in the city of Lille, among all women who had given birth during the 2014–2015 influenza season. Data were collected through a self-completed questionnaire and from medical files. The vaccination uptake was self-reported. Determinants of vaccination uptake were identified using logistic regression analysis.

**Results:**

Of the 2045 women included in the study, 35.5% reported that they had been vaccinated against influenza during their pregnancy. The principal factors significantly associated with greater vaccination uptake were previous influenza vaccination (50.9% vs 20.2%, OR 4.1, 95% CI 3.1–5.5), nulliparity (41.0% vs 31.3%, OR 2.5, 95% CI 1.7–3.7), history of preterm delivery < 34 weeks (43.4% vs 30.3%, OR 2.3, 95% CI 1.1–4.9), the mother’s perception that the frequency of vaccine complications for babies is very low (54.6% vs 20.6%, OR 1.1, 95% CI 0.5–2.2), the mother’s good knowledge of influenza and its vaccine (61.7% vs 24.4%, OR 3.1, 95% CI 2.2–4.4), hospital-based prenatal care in their first trimester of pregnancy (55.0% vs 30.2%, OR 2.1, 95% CI 1.2–3.7), vaccination recommendations during pregnancy by a healthcare worker (47.0% vs 2.7%, OR 18.8, 95% CI 10.0–35.8), receipt of a vaccine reimbursement form (52.4% vs 18.6%, OR 2.0, 95% CI 1.5–2.7), and information from at least one healthcare worker about the vaccine (43.8% vs 19.1%, OR 1.8, 95% CI 1.3–2.6).

**Conclusions:**

Our findings suggest that in order to increase flu vaccination compliance among pregnant women, future public health programmes must ensure cost-free access to vaccination, and incorporate education about the risks of influenza and the efficacy/safety of vaccination and clear recommendations from healthcare professionals into routine antenatal care.

## Introduction

Seasonal influenza is a common and contagious illness with an annual attack rate estimated at 5–10% in adults [1], pregnant women being at increased risks of morbidity and death [1], even those with no comorbidities [2].

Seasonal influenza vaccination during pregnancy reduces the risk of an influenza diagnosis by 50% [3]. It also confers effective protection up to the age of 6 months for newborns whose mother was vaccinated during pregnancy [4] with a reduction of 63% in influenza cases and of 29% in episodes of febrile respiratory illness [5]. A review of 15 years of surveillance data covering 750 million doses of the vaccine in the United States revealed no data that raised concerns about its safety in general population [6], neither for the foetus nor the mother, as showed by other studies [7–10]. As a result, the World Health Organization (WHO) [11], the American College of Obstetricians and Gynecologists and the Centers for Disease Control and Prevention (CDC) [12], recommend seasonal influenza vaccination for pregnant women, regardless of gestational age.

Several previous studies have identified factors affecting pregnant women’s decisions about whether to get a seasonal influenza vaccination [13–17]. Despite this, the vaccination coverage in pregnant women remains very low: 7% in France in 2016 [18], 45% in England in 2017 [19], and 37% in the US in 2017 [20] and lower than the Healthy People 2020 target of 80% [21]. Therefore, to explore why the vaccination coverage remains very low, we conducted one of the largest cohort studies on this topic to date to evaluate women motivations to be vaccinated or not. We also investigated a large number of possible determinants, in order to find strategies to improve seasonal influenza vaccination rate.

## Material and methods

### Study design and sampling method

We conducted a prospective single centre observational study during the 2014–2015 influenza season in a level-III University maternity unit in Lille, France, with an approximate birth rate of 5000 births/year. In France, women must obtain a prescription and a reimbursement form from their general practitioner or antenatal care provider, purchase the vaccine from a drugstore and the vaccine may then be administered during another appointment with the healthcare worker conducting the antenatal care or by a nurse at home. The vaccine is cost-free if the woman provides the drugstore with a reimbursement form. Eligible women for the study were all the women giving birth in our maternity unit and having received prenatal care during the 2014–2015 vaccination campaign between November 17, 2014, and June 5, 2015. The study excluded those younger than 18 years, or who did not speak French, or had a contraindication to the influenza vaccination, or refused to participate. For all participants written consent was obtained.

### Variables considered in our study

The outcome of interest was seasonal influenza vaccination uptake, reported by the pregnant women as part of a self-completed questionnaire. Data were collected from medical forms and from a self-completed paper questionnaire (see Additional file 1 and Additional file 2)offered by the clinical staff to all eligible women during their postpartum hospitalisation. Variables considered as possible determinants of vaccine uptake were

1. maternal sociodemographic characteristics: age, educational level and living or not with her partner;
2. maternal medical characteristics before pregnancy: pre-existing comorbidities for which influenza vaccination is indicated according to French guidelines (grouped into major categories: respiratory, cardiac, neurological, kidney-related, haematological and immune-related, diabetes, chronic liver disease, and obesity with Body Mass Index (BMI) ≥ 40 kg/m^2^ [22]), being vaccinated against influenza before this pregnancy, number of previous deliveries, history of preterm delivery before 34 weeks;
3. characteristics of the current pregnancy: smoking status, obstetrical complications defined as gestational diabetes, gestational hypertension, pre-eclampsia, HELLP syndrome, infections and foetal growth restriction;
4. antenatal care: the starting time of prenatal care at the hospital, the healthcare worker providing the prenatal care being a gynaecologist-obstetrician, general practitioner, hospital midwife, private midwife, profession of the healthcare worker recommending the vaccination, the provision of a reimbursement form for the vaccine;
5. maternal knowledge about influenza and its vaccine: frequency of influenza, knowledge of serious complications of influenza for mothers and their infants, the frequency of vaccine complications for mothers and their infants, knowledge about the recommendation of the vaccine in pregnancy.

Data about maternal sociodemographic characteristics, maternal medical characteristics before pregnancy, and characteristics of this pregnancy were extracted from the medical forms. Prenatal care and maternal knowledge about influenza and its vaccine were extracted from the self-completed questionnaire. The questionnaire was adapted from the questionnaire used by Yudin et al. to assess women’s knowledge of influenza and its vaccination [23]. We also created a “knowledge score” about this disease and its vaccine before the study with a multidisciplinary group of experts including obstetricians, infectious disease specialists, general practitioners, and statisticians from the potential responses to the self-completed questionnaire (see Additional file 3). The score ranged from 0 to 9 points and a woman was considered to have good knowledge when her score was higher than the last quartile of the score distribution, that is, a knowledge score greater than 5.4/9 in our study.

### Statistical analyses

To identify determinants associated with vaccination uptake, we conducted bivariate and multivariate logistic regression analysis. Dependent variables included in the regression model were those previously described as determinants of vaccine uptake in scientific literature or associated with vaccination uptake in bivariate analysis with a *p*-value less than 0.20. We have calculated variance inflation factor to check for multicollinearity and all the variables have a VIF < 2.0. In order to evaluate if the profession of the healthcare worker recommending the vaccination was a relevant factor, we conducted an analysis only on women to whom vaccination had been recommended during pregnancy. Percentages were compared using the chi-2 test or Fisher’s exact test, depending on the number of individuals. We calculated adjusted odds ratios (aORs) with their 95% confidence interval (95% CI). The difference was considered significant if *p* < 0.05. The analyses were performed with STATA software version 13.0.0 (Copyright 1985–2013 StataCorp LP, StataCorp, College Station, TX, USA).

Our study adheres to the STROBE guidelines. The CEROG (committee for ethics in research in gynaecology and obstetrics, n° CEROG OBS 2014-11-01) approved of this study.

### Results

**Fig. 1:**
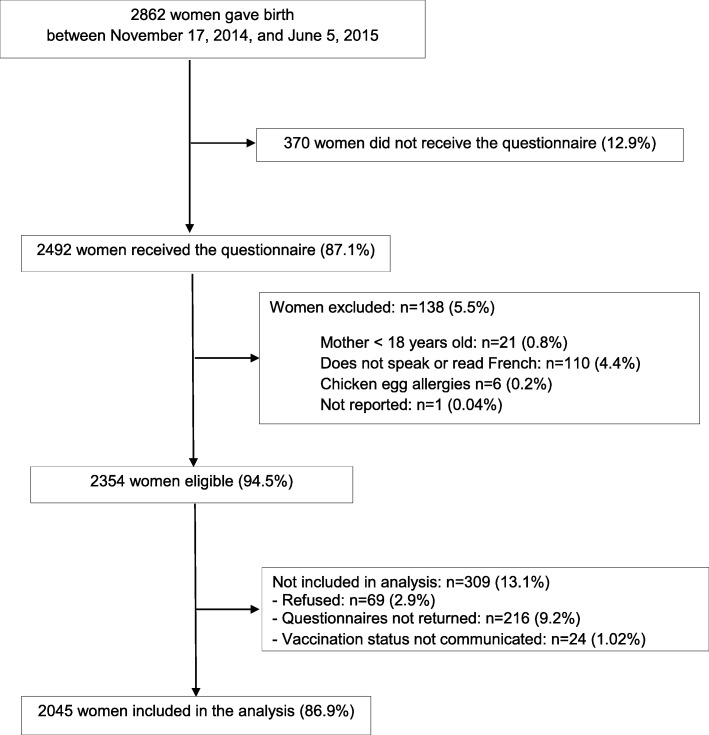
Strengthening the Reporting of Observational Studies in Epidemiology (STROBE) flow diagram of the participants in this study

Of the 2862 women who gave birth during the inclusion period, 370 did not receive the questionnaire (12.9%), 138 were excluded from the study (5.5%), 216 women received the questionnaire but did not return it (9.2%), and 24 women did not answer the question about their influenza vaccination. Therefore, 2045 women (86.9%) were included in the analysis (Fig. 1).

One third of the women questioned (35.5%) reported they had been vaccinated against seasonal influenza during their pregnancy. Table 1 presents the maternal factors associated with this vaccination. Women were vaccinated more often if they had at least one comorbidity (40.0%), if they had previously been vaccinated against influenza (50.9%), especially during a previous pregnancy (92.0%), and if they were nulliparous (41.0%). Higher vaccination rates were also observed for women who perceived influenza as a common disease (37.6%), or its vaccination as having a very low rate of complications for mothers (52.3%) or babies (54.6%), and when they had good knowledge about influenza (61.7%). Note that educational level and severe obesity (BMI > 40 kg/m^2^) were not associated with vaccination.

**Table 1:** Factors associated with uptake of influenza vaccination during pregnancy according to mothers’ characteristics (*n* = 2045)

	Vaccinated		
	n/N^a^	%	*p*^*¥*^
Total	725/2045	35.5	
Age			
< 25 years	97/306	31.7	0.49
≥ 25 and < 30 years	230/645	35.7	
≥ 30 and < 35 years	246/685	35.9	
≥ 35 years	151/408	37.0	
Educational level			
Primary	28/69	40.6	0.59
Secondary or technical	132/386	34.2	
Higher study	564/1588	35.5	
Lives with partner			
Yes	643/1803	35.7	0.56
No	81/240	33.7	
Smoked during pregnancy			
Yes	144/437	33.0	0.22
No	578/1602	36.1	
At least one comorbidity^b^	191/478	40.0	0.02
Respiratory	48/142	33.8	
Cardiac	28/74	37.8	
Neurological	36/93	38.7	
Nephrological	9/18	50.0	
Haematologic-immune	31/54	57.4	
Diabetes	16/30	53.3	
Chronic liver disease	10/21	47.6	
BMI ≥ 40 kg/m^2^	13/24	29.5	
Previous influenza vaccination			
Yes, outside pregnancy	279/548	50.9	< 0.001
Yes, during a previous pregnancy	185/201	92.0	
No	249/1235	20.2	
Number of previous deliveries			
0	358/874	41.0	< 0.001
1	225/704	32.0	
≥ 2	141/465	30.3	
History of preterm delivery < 34 weeks			
Yes	33/76	43.4	0.14
No	691/1967	35.1	
Obstetric complications^c^			
Yes	288/812	35.5	0.98
No	435/1228	35.4	
Perceived frequency of influenza in the general population			
Very low to low	49/180	27.2	0.02
Intermediate	217/631	34.4	
High	452/1202	37.6	
Perceived frequency of vaccine complications in pregnant women			
Very low	352/673	52.3	< 0.001
Low	154/509	30.3	
Intermediate	139/597	23.3	
High	52/141	36.9	
Perceived frequency of vaccine complications in babies			
Very low	375/687	54.6	< 0.001
Low	122/414	29.5	
Intermediate	128/621	20.6	
High	64/181	35.4	
Good knowledge of influenza^±^			
Yes	374/606	61.7	< 0.001
No	351/1439	24.4	

^a^Number of women vaccinated among the total number of women in the subclass

^b^Presence of at least one comorbidity that is an indication for influenza vaccination even outside of pregnancy according to the 2012 HAS guidelines [32]

^c^Gestational diabetes, hypertension, pre-eclampsia, HELLP syndrome, infections, other (anaemia, foetal growth restriction, etc.)

¥p value was calculated by Chi 2 test

±good knowledge of influenza was defined by a knowledge score > 5.4/9

The prenatal care factors associated with vaccination (Table 2) were hospital-based prenatal care in their first trimester of pregnancy (55.0%), having received a vaccination recommendation (47.0%), especially by a general practitioner (57.3%) or a midwife in private practice (54.3%), receipt of a vaccine reimbursement form (52.4%), or information from a healthcare worker (43.8%).

**Table 2:** Factors associated with uptake of influenza vaccination during pregnancy according to prenatal care (n = 2045)

	Vaccinated		
	n/N^a^	%	*p*^*¥*^
Total	725/2045	35.5	
Time at which prenatal care started			
First trimester	72/131	55.0	< 0.001
Second trimester	410/1106	37.1	
Third trimester	241/798	30.2	
Healthcare worker providing the prenatal care			
Assistant Chief Resident	50/129	38.8	0.80
Hospital staff physician	274,734	37.3	
Hospital staff midwife	322/894	36.0	
Intern	64/187	34.2	
Healthcare worker recommending vaccination			
Gynaecologist-Obstetrician	237/467	50.7	< 0.001
General practitioner	82/143	57.3	
Hospital staff midwife	229/571	40.1	
Midwife (in private practice)	25/46	54.3	
Several different professionals	76/158	48.1	
Occupational doctor, national health insurance	56/107	52.3	
Types of information received			
Recommendation for vaccination with a form for reimbursement	524/987	53.1	< 0.001
Recommendation for vaccination without a form for reimbursement	176/501	35.1	
No recommendation for vaccination but reimbursement form provided	3/19	15.8	
Neither proposal for vaccination nor reimbursement form	11/507	2.2	
Vaccination recommendation			
Yes	711/1514	47.0	< 0.001
No	14/528	2.7	
Receipt of a vaccine reimbursement form			
Yes	527/1006	52.4	< 0.001
No	187/1008	18.6	
Sources of information about influenza vaccination (multiple responses possible)			
At least one healthcare worker	604/1378	43.8	< 0.001
Not a healthcare worker^b^	117/611	19.1	

^a^Number of women vaccinated among the total number of women in the subclass

^b^All answers possible except healthcare workers: the media, discussion groups, family and friends, health authorities, and others

¥p value was calculated by Chi 2 test

Women motivations to be vaccinated or not are summarised in Table 3. The major motivation to be vaccinated was that the vaccine protects the baby (83%) and at the second place that the vaccine protects her (73%). A third of the vaccinated women claimed as motivation that they had received sufficient information about the benefits of the vaccine. However, there was a variety of reasons to not be vaccinated: some did not have enough information about the benefit and risk of the vaccine (32%), some were rather “against” vaccines in general (26%) and others were scared for the baby’s health (24%).

**Table 3:** Women motivations to be vaccinated or not against influenza

	n	%
Motivations to be vaccinated (*N* = 325)		
The vaccine protects me	529	73.0
The vaccine protects my baby	599	82.6
I have received sufficient information on the benefits of the vaccine	217	30.0
I am more “in favour” of vaccines in general	172	23.7
The vaccine is fully reimbursed	64	8.8
Other	23	3.2
Motivations not to be vaccinated (*N* = 1320)		
I did not know there was a vaccine	55	4.2
I was scared for my baby’s health	317	24.0
I was scared for my health	166	12.6
I did not have enough information about the benefits and risks	422	32.0
I am rather “against” vaccines in general	350	26.5
Other	387	29.3

On logistic regression analysis (Table 4), statistically significant determinants of vaccination were a previous influenza vaccination (50.9% vs 20.2%, OR 4.1, 95% CI 3.3–5.5), nulliparity (41.0% vs 30.3%, OR 2.5, 95% CI 1.7–3.7), history of preterm delivery < 34 weeks (43.4% vs 35.1%, OR 2.3, 95% CI 1.1–4.9), perception that the frequency of vaccine complications for babies is very low (54.6% vs 35.4%,OR 1.1, 95% CI 0.5–2.2), the mothers’ good knowledge of influenza and the vaccine (61.7% vs 24.4%,OR 3.1, 95% CI 2.2–4.4), hospital-based prenatal care in their first trimester of pregnancy (55% vs 30.2%, OR 2.1, 95% CI 1.2–3.7), vaccination recommendations (47.0% vs 2.7%, OR 18.8, 95% CI 10.0–35.8) and when this recommendation was done by a general practitioner (57.3% vs 50.7% for Gynaecologist-Obstetrician, OR 1.6 CI 1.0–2.8), receipt of a vaccine reimbursement form (52.4% vs 18.6%, OR 2.0, 95% CI 1.5–2.7), and having received information about the vaccine from at least one healthcare worker (43.8% vs 19.1%, OR 1.8, 95% CI 1.3–2.6).

**Table 4:** Logistical regression analysis of the factors associated with influenza vaccination uptake during pregnancy in this study (*n* = 1751)

	OR	aOR^a^	95% CI %^b^	*p*^*¥*^
Previous influenza vaccination				
No.	1	1		< 0.001
Yes, not during pregnancy	4.1	4.1	3.1–5.5	
Yes, in a previous pregnancy	45.8	43.9	22.8–84.4	
Number of previous deliveries				
≥ 2	1	1		< 0.001
1	1.1	1.6	1.1–2.4	
0	1.6	2.5	1.7–3.7	
History of preterm delivery < 34 weeks				
No.	1	1		0.02
Yes	1.4	2.3	1.1–4.9	
Perceived frequency of vaccine complications in babies				
High	1	1		0.005
Intermediate	0.3	0.9	0.5–1.5	
Low	0.2	0.5	0.3–0.9	
Very low	0.4	1.1	0.5–2.2	
Good knowledge of influenza				
No.	1	1		< 0.001
Yes	5.0	3.1	2.2–4.4	
Hospital-based prenatal care beginning				
Third trimester	1	1		0.02
Second trimester	1.4	1.2	0.9–1.6	
First trimester	2.8	2.1	1.2–3.7	
Vaccination recommendation				
No.	1	1		< 0.001
Yes	32.5	18.8	10.0–35.8	
Receipt of a vaccine reimbursement form				
No.	1	1		< 0.001
Yes	4.8	2.0	1.5–2.7	
Profession of the healthcare worker offering the vaccination^£^				
Gynaecologist-Obstetrician	1	1		0.05
General practitioner	1.3	1.6	1.0–2.8	
Hospital staff midwife	0.6	0.9	0.6–1.3	
Midwife (in private practice)	1.1	2.2	0.9–5.1	
Several different professionals	0.9	1.2	0.7–1.9	
Occupational doctor, national health insurance	1.1	1.7	1.0–2.9	
Sources of information about influenza vaccination (multiple responses possible)				
No healthcare worker^c^	1	1		< 0.001
At least one healthcare worker	3.3	1.8	1.3–2.6	

^a^Adjusted odds ratio: determined by multivariate logistic regression of influenza vaccination for the variables with a *p*-value < 0.20. The variables not significantly associated with vaccination (*p* > 0.05) are not presented: the presence of at least one comorbidity^b^, perceived frequency of influenza, and perceived frequency of vaccine complications in mothers

^b^95% confidence interval

^c^All answers possible except healthcare workers: the media, discussion groups, family and friends, health authorities, and others

¥p value was calculated by multivariate logistic regression analyses

£ Only women who had received a vaccination recommendation were analysed (*n* = 1300)

## Discussion

Our study examined the potential determinants of the influenza vaccination uptake amongst pregnant women in a single centre in France.

Overall, our findings highlight the importance of the healthcare worker in vaccination uptake. Indeed, vaccination recommendation by a healthcare provider strongly influence vaccination uptake (aOR 19). In addition to making a recommendation, the influence of healthcare worker was also vital in educating women about the influenza and the vaccine and providing reimbursement form.

Indeed, among factors that may be modified to improve the vaccination rate, we found, similar to other authors, that several are related to knowledge and perception of influenza, its vaccine and its potential complications [13–15, 17, 23, 24]. Moreover, our study found that protecting the baby against influenza was the leading motivation for vaccination among those pregnant women who were vaccinated (83%). In a systematic review of the literature on the subject, 41% of the articles studied found that vaccine safety was a major concern among pregnant women, for all vaccines combined [25]. Furthermore, our study demonstrates the role of healthcare professionals as an essential source of information for the pregnant women who are vaccinated. Inversely, sources of information such as television, radio, the print media, the internet, family and friends are associated with lower vaccination rates in our study and in the literature [13, 25, 26]. Conversely, good knowledge of influenza and its vaccine was closely associated with vaccination among the women questioned in our study. This should provide incentives to implement measures to improve the quality of information provided to women by healthcare worker [15, 27].

Concerning the major impact of the recommendation and provision of the reimbursement form by healthcare worker, our results are corroborated by the data from the literature: an analysis by the CDC for the 2016–2017 influenza season in the US showed that the vaccination rate among pregnant women reached 70.5% among women whose providers recommended and offered the vaccination, 43.7% when the vaccine was recommended but not offered, and 14.8% when it was neither recommended nor offered [20] (53%, 35 and 2% in our study).

In France, women must obtain a prescription from their GP or antenatal care provider, purchase the vaccine, and subsequently attend again to receive the vaccine. This process may be a significant barrier to the vaccine uptake. Several experiments are in process in France to study if getting the vaccine without prescription and injecting it at the same time in the drugstore can improve the vaccination coverage.

We found several other factors associated with increased vaccination rate but not easily modifiable by a public health program such as nulliparity [15, 17], history of preterm delivery or hospital based prenatal care in their first trimester of pregnancy [15, 28].

So, our study highlights the importance of incorporating education about the risk of flu and the safety/efficacy of vaccination into routine antenatal care. The pregnant women need to know that the vaccine protects them and their newborns from influenza infection and that there are very few vaccine complications for them and their babies. Our study suggests that this simple message delivered by a healthcare worker can improve the vaccination uptake.

### Implications for future practice and research

Our study suggests that in order to increase influenza vaccine compliance among pregnant women, two principal actions should guide prenatal care policies: information about influenza and its vaccine by healthcare workers and cost-free access to the vaccine. As the role of professionals appears central, it would be useful to conduct a study in order to assess their knowledge and opinions about this vaccination and to help them to improve their practice by incorporating education about influenza vaccination during their routine antenatal care.

### Strengths and limitations

On the one hand, the prospective nature of our study, the number of women included, the high participation rate (87%), and the use of a self-completed questionnaire enabled us to limit the potential for bias as much as possible. On the other hand, its single-centre nature could have induced recruitment bias, and the responses cannot be taken as representative of all pregnant women. Another limitation might be that our participants were all recruited from a university hospital, and that our sample may therefore be missing subsets of the population that tend to be more anti-vaccination or receive less education, so future studies might benefit from recruiting over a wider geographical area and from different types of sites. Finally, vaccination status was reported by the women and there is therefore potentially susceptible to reporting bias, which has been partially corrected by checking the women’s medical records.

## Conclusions

Although the World Health Organization has recommended influenza vaccination for all pregnant women since 2012, only one third of the mothers in our study were vaccinated. Our study highlights that in order to increase influenza vaccination compliance among pregnant women, two principal actions should guide prenatal care policies: information about influenza and its vaccine be routinely given by healthcare workers and cost-free access to the vaccine.

## Supplementary information

**Additional file 1.** Questionnaire in French

**Additional file 2.** Questionnaire in English

**Additional file 3.** Knowledge score, a.File explaining how the knowledge score was constructed

## Data Availability

All datasets analysed during this study are presented in an additional supporting file.

## Abbreviations

95% CI: 95% confidence interval
aORs: Adjusted odds ratios
BMI: Body Mass Index
CDC: Centers for Disease Control and Prevention
CEROG: Committee for ethics in research in gynaecology and obstetrics
HELLP syndrome: Haemolysis, Elevated Liver enzymes and Low Platelet count syndrome
OR: Odds ratio
US: The United States
WHO: World Health Organization
