# Supplementary material for: Determinants of influenza vaccination uptake in pregnancy: a large single-Centre cohort study
Source: BMC Pregnancy Childbirth. 2019 Dec 19;19:510. doi: 10.1186/s12884-019-2628-5 (PMC6924067; doi:10.1186/s12884-019-2628-5)
Supplement: Supplementary file 1 — Additional file 1. Questionnaire in French [file 12884_2019_2628_MOESM1_ESM.docx]

1. According to you, the flu is a disease: (for each proposal, circle the number that corresponds to your opinion)

Very rare Very frequented

0 1 2 3 4 5 6 7 8 9

If answer <5 (very low to low): 1pt.

Never serious Always serious

0 1 2 3 4 5 6 7 8 9

If answer <3 (very low): 1 pt.

If answer ≥3 and <5 (low): 1pt.

1. Do you think flu can cause serious complications during pregnancy in the mother?

If answer yes: 1pt.

1. Do you think that flu can cause serious complications during pregnancy in the baby?

If answer yes: 1pt.

1. According to you, vaccination against influenza during pregnancy is:

If answer Might be useful: 0.5 pt.

If answer Definitely useful: 1 pt.

1. According to you, vaccination against influenza during pregnancy is:

If answer obligatory: 0.5 pt.

If answer recommended: 1 pt.

1. According to you, the flu vaccine can cause complications for the mother:

*(circle the number that corresponds to your opinion)*
Very rare Very frequent

0 1 2 3 4 5 6 7 8 9

If answer ≥ 5 and <7: 1 pt.

Never serious Always serious

0 1 2 3 4 5 6 7 8 9

If answer <3 (very low): 1pt

1. According to you, the flu vaccine can cause complications in the baby:
   Very rare Very frequent

0 1 2 3 4 5 6 7 8 9

If answer < 3: 1pt.

Never serious Always serious

0 1 2 3 4 5 6 7 8 9

If answer <3 (very low): 1pt.
