# Supplementary material for: Determinants of influenza vaccination uptake in pregnancy: a large single-Centre cohort study
Source: BMC Pregnancy Childbirth. 2019 Dec 19;19:510. doi: 10.1186/s12884-019-2628-5 (PMC6924067; doi:10.1186/s12884-019-2628-5)
Supplement: Supplementary file 2 — Additional file 2. Questionnaire in English [file 12884_2019_2628_MOESM2_ESM.doc]

G2014/1/ _ _ _ _

Name

First name

Birth date

G2014/1/ _ _ _ _

1. In order to participate at this study, we need to know some information :

- Are you over 18 years old ?🞎 yes no 🞎
- Do you speak French? 🞎 yes no 🞎

- do you read French?🞎 yes 🞎 with difficulty 🞎 not at all

- are you allergic to egg proteins? 🞎 no yes 🞎

Answer the 4 questions THEN if a gray box checked in question a): STOP

- Do you agree to fill out this questionnaire? 🞎 yes no 🞎

1. What is your level of education?

- Primary school 🞎 secondary or technical 🞎 higher

1. When did you start your pregnancy monitoring in Jeanne de Flandre hospital?

- 1^st^ trimester 🞎 2^nd^ trimester 🞎 3^rd^ trimester

1. What is the name of the person who followed you at Jeanne de Flandre hospital?……………………………….

1) According to you, the flu is a disease: (for each proposal, circle the number that corresponds to your opinion)

Very rare Very frequented

0 1 2 3 4 5 6 7 8 9
Never serious Always serious

0 1 2 3 4 5 6 7 8 9

1. Do you think flu can cause serious complications during pregnancy in the mother?

- Yes 🞎 No 🞎 I don't know
- If yes, can you give an example? …………………………………………………………………………………………

1. Do you think that flu can cause serious complications during pregnancy in the baby?

- Yes 🞎 No 🞎 I don't know
- If yes, can you give an example? ………………………………………………………………………………………….

1. According to you, vaccination against influenza during pregnancy is:

- Contraindicated 🞎 Unnecessary 🞎 Might be useful 🞎 Definitely useful

1. According to you, vaccination against influenza during pregnancy is:

- Obligatory 🞎 Neither obligatory nor recommended
- Recommended by health authorities 🞎 I don't know

1. Have you ever been vaccinated against influenza?

- Yes, outside pregnancy 🞎 No
- Yes, during a previous pregnancy 🞎 I don't know

1. What were your sources of information regarding influenza vaccination? (many possible responses)

- Healthcare worker (doctor, midwife, nurse, pharmacist ...)
- Media (radio, television, newspapers and magazines, internet, advertising posters, …)
- Discussion forums (Chat, associations of people ...)
- Entourage (family, friends ...)
- Official health authorities (HAS, Inpes, Ansm ....)
- Other : ………………………………………………………………………………………………………………………………..............

1. Among these sources, specify the one that motivated your decision (to vaccinate or not)? …………………………..

**TOURNEZ SVP**

1. During this pregnancy, have you been offered vaccination against influenza?

- Yes 🞎 No
- If so, who proposed it to you?

🞎 Gynecologist Obstetrician of Jeanne of Flandre hospital 🞎 General practitioner

🞎 Jeanne de Flandre midwife 🞎 Liberal midwife

🞎 Other : …………………………………………………………………………………………………………………………………..

1. During the pregnancy, were you given the form of reimbursement of the vaccination by social insurance (free of charge)?

- Yes 🞎 No

1. Have you been vaccinated against the flu during this pregnancy?

- Yes 🞎 No 🞎 I don't know

**☞If yes**,

- by whom ? ………………………………………………………………………………………………………
- at what time of pregnancy did you perform the vaccination ?
- 1^st^ trimester (0 -3 months) 🞎 2^nd^ trimester (4-6 months) 🞎 3^rd^ trimester (7-9 months)
- What were your arguments? (many possible responses)
- The vaccine protects me
- The vaccine protects my baby
- I have received sufficient information on the benefits of the vaccine
- I am more “in favor” of vaccines in general
- The vaccine is fully reimbursed
- Other : ……………………………………………………………………………………………………………………………….

**☞If no,**

- What were your reservations? (many possible responses)
- I did not know there was a vaccine
- I was scared for my baby's health
- I was scared for my health
- I did not have enough information about the benefits and risks
- I am rather "against" vaccines
- Other : ……………………………………………………………………………………………………………………………….

1. Which person helped you most in making your choice?

🞎 General practitioner 🞎 liberal obstetrician/midwife 🞎 hospital obstetrician/midwife 🞎 Friend/Family

🞎 Other

1. According to you, the flu vaccine can cause complications for the mother:

*(circle the number that corresponds to your opinion)*
Very rare Very frequent

0 1 2 3 4 5 6 7 8 9

Never serious Always serious

0 1 2 3 4 5 6 7 8 9

1. According to you, the flu vaccine can cause complications in the baby:
   *(circle the number that corresponds to your opinion)*
   Very rare Very frequent

0 1 2 3 4 5 6 7 8 9

Never serious Always serious

0 1 2 3 4 5 6 7 8 9

1. If you were not vaccinated, do you think to do it now?

🞎 Yes 🞎 No

**A THANK YOU FOR YOUR PARTICIPATION!**
