# Supplementary material for: Determinants of influenza vaccination uptake in pregnancy: a large single-Centre cohort study
Source: BMC Pregnancy Childbirth. 2019 Dec 19;19:510. doi: 10.1186/s12884-019-2628-5 (PMC6924067; doi:10.1186/s12884-019-2628-5)
Supplement: Supplementary file 3 — Additional file 3. Knowledge score, a.File explaining how the knowledge score was constructed [file 12884_2019_2628_MOESM3_ESM.doc]

G2014/1/ _ _ _ _

Etiquette patiente

QUESTIONNAIRE PATIENTES SUITE DE NAISSANCE

G2014/1/ _ _ _ _

1. Pour pouvoir participer à cette étude, nous devons connaître certaines informations :

- avez-vous plus de 18 ans ? 🞎 oui non 🞎

- parlez-vous français ? 🞎 oui non 🞎

- lisez-vous le français 🞎 oui 🞎 difficilement 🞎 pas du tout

- êtes-vous allergique aux protéines de l’œuf ? 🞎 non oui 🞎

**Répondre aux 4 questions PUIS**

**si une case grise cochée à la question a) : STOP**

- êtes-vous d’accord pour remplir ce questionnaire ? 🞎 oui non 🞎

1. Quel est votre niveau d’études ?

- Primaire 🞎 Secondaire ou technique 🞎 Baccalauréat ou supérieur

1. Quand avez-vous débuté votre suivi de grossesse à Jeanne de Flandre ?

- 1^er^ trimestre 🞎 2^ème^ trimestre 🞎 3^ème^ trimestre

1. Quel est le nom de la personne qui vous a suivie à Jeanne de Flandre (au moins 2 fois) ?……………………………….
2. Selon vous, la ***grippe*** est une maladie : *(pour chaque proposition, entourez le chiffre qui correspond à votre opinion)*

Très rare Très fréquente
 0 1 2 3 4 5 6 7 8 9
Jamais grave Toujours grave

0 1 2 3 4 5 6 7 8 9

1. Pensez-vous que la grippe peut entraîner des ***complications graves*** pendant la grossesse ***chez la mère*** ?

- Oui 🞎 Non 🞎 Je ne sais pas
- Si oui, pouvez-vous donner un exemple ? …………………………………………………………………………………………

1. Pensez-vous que la grippe peut entrainer des ***complications graves*** pendant la grossesse ***chez le bébé*** ?

- Oui 🞎 Non 🞎 Je ne sais pas
- Si oui, pouvez-vous donner un exemple ? ………………………………………………………………………………………….

1. Selon vous, la ***vaccination*** contre la grippe pendant la ***grossesse*** est :

- Contre indiquée 🞎 Inutile 🞎 Peut être utile 🞎 Certainement utile

1. Selon vous, la ***vaccination*** contre la grippe pendant la ***grossesse*** est :

- Obligatoire 🞎 Ni obligatoire, ni recommandée
- Recommandée par les autorités de santé 🞎 Je ne sais pas

1. Avez-vous déjà été ***vaccinée contre la grippe*** ?

- Oui, en dehors d’une grossesse 🞎 Non
- Oui, lors d’une précédente grossesse 🞎 Je ne sais pas

1. Quelles ont été vos ***sources d’information*** concernant la ***vaccination*** contre la grippe ? (*plusieurs réponses possibles)*

- Professionnels de santé (médecin, sage-femme, infirmier, pharmacien…)
- Médias (radio, télévision, journaux et magazines, internet, affiches publicitaires, …)
- Forums de discussion (Chat, associations de personnes…)
- Entourage (famille, amis…)
- Autorités officielles de santé (HAS, Inpes, Ansm….)
- Autres : ………………………………………………………………………………………………………………………………..............

1. Parmi ces ***sources***, précisez ***celle*** qui a motivé votre décision (se vacciner ou non) ? ……………………………………..

**TOURNEZ SVP**

1. ***Au cours de cette grossesse***, vous a-t-on ***proposé*** la vaccination contre la grippe ?

- Oui 🞎 Non
- Si oui, qui vous l’a proposée ?

🞎 Gynécologue Obstétricien de Jeanne de Flandre 🞎 Médecin généraliste

🞎 Sage-femme à Jeanne de Flandre 🞎 Sage-femme libérale

🞎 Autre : …………………………………………………………………………………………………………………………………..

1. Pendant la grossesse, vous a-t-on remis le ***formulaire de remboursement*** de la vaccination par la sécurité sociale (bon de gratuité)?

- Oui 🞎 Non

1. Avez-vous été ***vaccinée*** contre la grippe au cours de ***cette grossesse*** ?

- Oui 🞎 Non 🞎 Je ne sais pas

**☞Si oui**,

- par qui ? ………………………………………………………………………………………………………
- à quel moment de la grossesse avez-vous effectué la vaccination ?
- 1^er^ trimestre (0 -3 mois) 🞎 2^ème^ trimestre (4-6 mois) 🞎 3^ème^ trimestre (7-9 mois)
- quels ont été vos arguments ? *(plusieurs réponses possibles)*
- Le vaccin me protège
- Le vaccin protège mon bébé
- J’ai reçu des informations suffisantes sur les bénéfices du vaccin
- Je suis plutôt « pour » les vaccins en général
- Le vaccin est remboursé intégralement
- Autre : ……………………………………………………………………………………………………………………………….

**☞Si non,**

- quelles ont été vos réticences ? *(plusieurs réponses possibles)*
- Je ne savais pas qu’il existait un vaccin
- J’ai eu peur pour la santé de mon bébé
- J’ai eu peur pour ma santé
- Je n’ai pas eu assez d’informations sur les bénéfices et les risques
- Je suis plutôt « contre » les vaccins
- Autre : ……………………………………………………………………………………………………………………………….

1. Quelle ***personne*** vous a surtout aidée à faire ***votre*** ***choix*** ?

🞎 Médecin traitant 🞎 Gynéco/sage-femme de ville 🞎 Gynéco/sage-femme maternité 🞎 Ami/famille 🞎 Autre

1. Selon vous, le ***vaccin*** antigrippal peut entraîner, ***chez la mère***, des ***complications*** :

*(entourez le chiffre qui correspond à votre opinion)*
Très rares Très fréquentes

0 1 2 3 4 5 6 7 8 9

Jamais graves Toujours graves

0 1 2 3 4 5 6 7 8 9

1. Selon vous, le ***vaccin*** antigrippal peut entraîner, ***chez le bébé***, des ***complications*** :
   *(entourez le chiffre qui correspond à votre opinion)*
   Très rares Très fréquentes

0 1 2 3 4 5 6 7 8 9

Jamais graves Toujours graves

0 1 2 3 4 5 6 7 8 9

1. Si vous n’étiez pas vaccinée, ***pensez-vous le faire maintenant*** ?

🞎 Oui 🞎 Non

**UN GRAND MERCI POUR VOTRE PARTICIPATION !**
